# Evaluation of fluorescent dyes to measure protein aggregation within mammalian cell culture supernatants

**DOI:** 10.1002/jctb.5519

**Published:** 2018-01-05

**Authors:** Sheun Oshinbolu, Rachana Shah, Gary Finka, Mike Molloy, Mark Uden, Daniel G Bracewell

**Affiliations:** ^1^ Dept of Biochemical Engineering University College London London UK; ^2^ GlaxoSmithKline Stevenage Herts UK

**Keywords:** monoclonal antibodies, CHO cells, aggregation, fluorescence, extrinsic dyes

## Abstract

**BACKGROUND:**

A current challenge in bioprocessing is the ability to analyse critical quality attributes such as aggregation without prior purification. This study evaluated the use of fluorescent dyes (Bis‐ANS, SYPRO Orange, Thioflavin T and ProteoStat) to characterise mAb aggregates in Chinese hamster ovary clarified cultures.

**RESULTS:**

The null and mAb culture supernatants showed an increase in fluorescence intensity over the duration of the culture. The null cultures on day 14 saw a rapid increase in fluorescence intensity; day 10 to day 14, Bis‐ANS and Thioflavin T had average increases of 21% and 48%, respectively, whereas ProteoStat and SYPRO Orange showed an average increase of 60%. Higher fluorescence intensity on day 14 with the null cultures, also correlated with loss of viability.

**CONCLUSION:**

Fluorescent dyes are not a specific indicator of mAb aggregation, but rather an indicator of overall protein aggregation or high molecular weight species. SYPRO Orange was more sensitive at detecting very large molecular weight species and ProteoStat seemed better suited to smaller aggregates. Although the assay cannot be used to measure mAb aggregates in cell culture, it could be used to aid cell line selection in maximising viabilities and minimising the amount of aggregates. © 2017 The Authors. *Journal of Chemical Technology & Biotechnology* published by John Wiley & Sons Ltd on behalf of Society of Chemical Industry.

## INTRODUCTION

The development process of monoclonal antibodies (mAbs) is highly regulated due to the 10^8^ possible variants.^1^ To ensure high quality drugs are consistently produced, biopharmaceutical companies are becoming more risk adverse and hence moving towards characterising product quality earlier in development. One approach, ‘Quality‐by‐Design’ (QbD), enables confidence and predictability to be built into a process. QbD starts with identifying critical quality attributes (CQAs) which can be categorised as product or process related. Product related impurities are molecular variants of the product such as aggregates, incorrect glycosylation, charge variants, oxidation and fragmentation. Process related impurities are a result of the process environment introducing additional components to the product e.g. DNA, host cell proteins (HCP) and viruses. CQAs are often considered to have an effect on safety, activity and clearance.^2^ For mAbs, companies typically aim to have <5% high molecular weight species (HMW), <100 ppm HCP and <10 ng/dose DNA, although in reality, these limits are case‐by‐case dependent.^3^ Identifying CQAs allows one to control variability by understanding the impact of materials and process conditions on product quality.

One CQA in particular, product aggregation, can influence production, activity and safety.^4^ Aggregates can be difficult to characterise even after purification due to differences in size, structure, charge, solubility and mechanisms of formation. Not one single analytical technique can provide detailed aggregation characterisation as techniques vary in throughput, detection limit, sensitivity and accuracy. In bioreactors, the presence of HCPs, DNA, cells and other cellular impurities can interfere with measurements, hence aggregation characterisation is usually carried out on candidate molecules after purification. Although purification is possible, it results in longer timelines, increased costs/resources and key components (e.g. large aggregates) may be removed, thus ultimately providing data that may not be a true representation of the cell culture.

In the bioreactor, mAbs are exposed to many different physical (e.g. temperature and pH) and chemical stresses (e.g. oxidation and deamidation) that can cause conformational changes. The push for higher titres to reduce the cost of manufacture has elevated the risk of aggregation in the bioreactor due to the concentration‐dependent nature of aggregates. Dengl *et al*.^5^ showed that HMW aggregates present in the bioreactor tend to go undetected as they are removed in recovery/filtration steps and are only noticeable by increases in turbidity. Although, there has been significant progress to maximise titres, cell counts and viability, there is little understanding of how changes in the upstream conditions impact product aggregation in the bioreactor. Hence, there is a need to develop analytical assays that can measure aggregation in a complex multi‐component environment, which will save time and resources and aid in screening and selecting cell lines against product quality.

One possible technique is fluorescence spectroscopy which has been used for aggregation detection. Fluorescence signal is sensitive to solvent polarity, viscosity and temperature. Intrinsic fluorescence is derived from naturally fluorescent amino acids (e.g. tryptophan), whereas extrinsic fluorescence comes from the addition of fluorescent dyes. Fluorescence is measured by exciting a dye with a laser/lamp light which is absorbed by the dye, and lifts the electrons of the dye to a higher excited state.^6^ The electrons eventually relax back down to ground state by fluorescence emission. Upon interacting with protein aggregates, an increase in fluorescence intensity is usually accompanied with a blue or red shift of the peak maximum. A blue shift indicates the dye is in a hydrophobic environment, whereas a red shift indicates the dye is in a hydrophilic environment.

1‐anilinonaphthalene‐8‐sulfonate (ANS) and 4,4′‐bis‐1‐anilinonaphthalene‐8‐sulfonate (Bis‐ANS), Nile Red and SYPRO Orange are the most frequently used extrinsic dyes for aggregate characterisation.^7^ ANS and its dimeric form Bis‐ANS, have been used since the 1950s for protein characterisation. Bis‐ANS and ANS hardly fluoresce in aqueous environments but strongly fluoresce when interacting with hydrophobic sites. Hydrophobic interactions and electrostatic interactions have both been discussed as the binding mechanism of ANS to proteins.^8^ For Bis‐ANS, hydrophobic interactions are seen as the most dominant.^9^ However due to the larger size of Bis‐ANS, fluorescence can be inhibited by steric hindrance.

Fluorescence can also be used for visualisation of protein aggregates with microscopy^10^, ^11^ and to monitor protein folding/unfolding.^12^ The main application of SYPRO Orange was for staining SDS PAGE gels and western blots. It is now known to display high sensitivity to structurally altered/aggregated IgG structures compared with the native form.^13^ This sensitivity results in an increased fluorescence with increasing availability/presence of hydrophobic areas of unfolded proteins.

Thioflavin T (ThT) is in a specific class of dyes called molecular rotors, which have significant increases in fluorescence due to a decrease in torsional relaxation of molecules.^14^ In solution, molecular rotors rotate freely, however changes to the micro‐environment constrict the dye's movement, resulting in fluorescence. ThT fluorescence is affected more by changes in the solvent, viscosity and rigidity of the microenvironment than by polarity.^6^ It is mostly used to quantify amyloid fibrils which are filamentous protein aggregates about 10 nm width and 0.1 to 10 µm length and predominantly beta sheet secondary structure.^15^ Vetri *et al*. showed selectivity of ThT to fibrils and ANS to hydrophobic amorphous aggregates.^16^

A novel protein aggregation dye, ProteoStat by Enzo Life Science, is also a molecular rotor. ProteoStat works in a way such that in the absence of protein aggregates or at low viscosities, the dye spins in solution with no fluorescence. In the presence of aggregates, the dye slips into the exposed cavities of aggregated protein, thereby causing the dyes rotation to be constrained, resulting in fluorescence. It is currently the only commercial dye marketed for protein aggregate detection for visible to sub‐visible aggregates.

Even though there is a considerable amount of work in the literature using extrinsic dyes to measure aggregation, the focus has mostly been on the use of dyes on purified samples with the exception of Paul and Hesse.^17^ They suggested that Bis‐ANS is a suitable dye for measuring aggregates in cell cultures supernatants upon measuring aggregated mAb after 120 h culture. Paul and Hesse^17^ measured samples that had >65% mAb aggregates, however those working in process development will often aim to measure below 5% aggregates post‐purification as most mAbs tend to fall within this range to comply with regulatory guidelines. This will require a dye to be sensitive enough to detect aggregates in a sample which is largely monomeric mAb. In addition, as cell cultures were only run for up to 300 h (12 days), it would be interesting to see the impact of later harvest time points (>12 days) and elevated HCP levels, on dye fluorescence.

In this work, we investigated the suitability of extrinsic dyes to detect less than 10% mAb aggregates in Chinese hamster ovary (CHO) cell culture supernatants at varying culture time points up to day 14. The four dyes, Bis‐ANS, SYPRO Orange, ThT and ProteoStat, were evaluated to see which is the most sensitive. To assess the potential of the four dyes for mAb aggregate detection in CHO cell cultures, first the dyes were evaluated in buffer with thermally stressed mAb aggregates. Two of the dyes were then applied to CHO cell culture supernatants of mAb producing and non‐mAb producing cell lines to see the impact of HCP levels on fluorescence. We also characterised the size of aggregates the dyes may be binding to using size exclusion chromatography (SEC) with fluorescence detectors.

## MATERIALS AND METHODS

### Monoclonal antibody

IgG1 monoclonal antibody (mAb A and mAb B) produced in CHO cells were used in this study. The mAbs were cultured in proprietary chemical defined serum free media with mAb A produced in shake flasks and mAb B produced in a bioreactor. The culture was harvested at day 14 with viabilities and cell densities of 95% and 18×10^6^ cells mL^‐1^ and 76% and 4×10^6^ cells mL^‐1^ for mAb A and mAb B, respectively. The isoelectric point (pI) of IgG mAb A and B were 8.4 and 9.5, respectively. MAb A was used for experiments with purified mAb and mAb B was used for experiments with mAb in clarified cell culture supernatant. MAb A was protein A purified using MabSelect SuRe™ (GE Healthcare, Uppsala, Sweden) with a 185 mL column (23 × 3.2 cm) using an AKTA Avant system. After purification, mAb A was neutralised to pH 7.5 ± 0.1 and filtered using a 500 mL 0.22 µm filter system (Corning, New York). The purified mAb A was then dialysed into 50 mmol L^‐1^ sodium acetate pH 5.5 for storage at 4 °C. The CHO cell culture producing mAb B was sampled on day 1, 3, 7, 10 and 14. The mAb B samples were then spun down at 4000 rpm for 10 min, followed by 0.22 µm filtration (Millipore, Cork, Ireland) to remove cell debris remnants prior to assay measurements.

### Creation of aggregates

In order to generate aggregates to test the assay, mAb A was subjected to thermal stress. MAb A (11 mg mL^‐1^) was stressed at 60 °C for 72 h in an incubator and then 0.22 µm filtered. The stressed mAb A was used to assess the sensitivity of the dyes by spiking different amounts of stressed mAb into wells of a 96 well plate containing unstressed mAb A. As the stressed stock of mAb A was quantified to have 20% aggregated mAb A by SEC, the dye assay was used to detect varying percentages of spiked aggregates ranging from 0–20%.

### SEC analysis

Samples were analysed using SEC (injection volume 10 µL) with Agilent 1100. The column used was a TSKgel G3000SWXI (7.8 × 300 mm) from TOSOH Bioscience with a running buffer composed of 100 mmol L^‐1^ sodium phosphate (monobasic), 400 mmol L^‐1^ sodium chloride, pH 6.7. The flow rate was 1.0 mL min^‐1^ and protein was detected using UV detectors at 214 nm and 280 nm.

For SEC combined with fluorescence detection (Agilent 1100) with the dyes, injection of samples was increased to 50 µL to strengthen the signal. The same amount of dye used in the plate assay was added to the samples before injection into the column. Samples containing SYPRO Orange were excited at 495 nm and the emission read at 590 nm. Samples containing ProteoStat were excited at 530 nm and emission read at 605 nm.

### Plate based dye aggregation assay

50 µmol L^‐1^ Bis‐ANS, 5X SYPRO Orange, 3 µmol L^‐1^ ProteoStat and 50 µmol L^‐1^ ThT were the final concentration in each well. ThT (Sigma Aldrich, Bangalore, India) and Bis‐ANS (Sigma Aldrich, Steinhelm, Germany) were solubilised into MilliQ water. SYPRO Orange (Life Technologies, Eugene, Oregon) was diluted using MilliQ water and ProteoStat (Enzo Life Sciences, Farmingdale, New York) was prepared based on the manual protocol. Concentration of mAb A was kept to 1 mg mL^‐1^ when using stressed mAb (mAb in cell culture supernatants had varying titres) with a final volume of 200 µL in each well (n = 3). The excitation and emission wavelengths (ex/em) for each dye were as follows: Bis‐ANS 390/450–600 nm, SYPRO Orange 495/550–700 nm, ProteoStat 530/560–700 nm and Thioflavin 430/460–600 nm. The assay was measured using a 96 well clear flat‐bottom micro plate (Corning, Kennebunk, Maine) with TECAN Infinite M200 series. The following parameters were used for fluorescence measurement: bottom reading, integration time 20 µs and 25 flashes. The data were analysed using OriginPro and smoothed using Savitzky–Golay smoothing filter with a polynomial of 2 and a smoothing factor of 15.

### Cell culture samples

The null cell line (CHO‐K1A) was used to obtain time‐course cell culture supernatant samples in the absence of mAb. CHO‐K1A was cultured in proprietary chemical defined serum free media in three 600 mL shake flasks. The shakes flasks were sampled on day 0, 3, 7, 10, 14 and harvested at day 14 with viabilities and cell densities ranging from 53–85% and 11–18×10^6^ cells mL^‐1^, respectively. Cells were also spun down at 4000 rpm for 10 min followed by 0.22 µm filtration.

### Protein and HCP concentration

Pierce Coomassie Plus (Thermo Scientific, Rockford, Illinois) was used to measure the total protein concentration in cell culture supernatant samples. Samples acquired on and before day 3 of culture were measured according to supplier's micro–micro‐plate protocol due to lower concentrations of protein. Samples acquired after day 3 of culture were measured with the micro‐plate protocol. A GlaxoSmithKline in‐house sandwich ELISA was used to measure the concentration of HCP in the cell culture supernatants.

## RESULTS AND DISCUSSION

**Figure 1:**
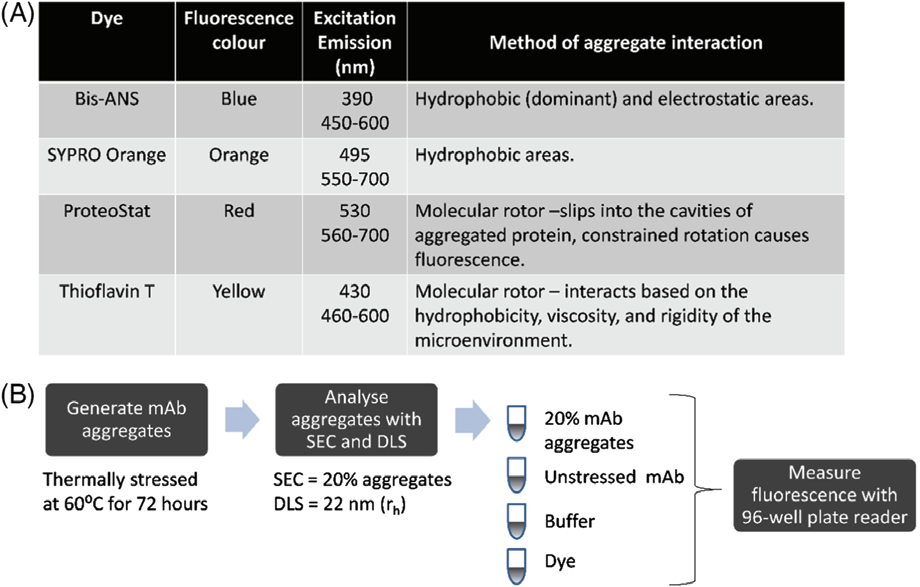
Selected dyes and spiking experiment method. (A) Aggregation detection mechanism of each dye. Table shows the excitation and emission conditions used in this study and the method by which each dye interacts with aggregates. (B) Spiking experiment method to generate aggregates for the spiking experiment. The aggregates were then measured by SEC and DLS and shown to have 20% aggregates with a hydrodynamic radius of 22 nm. To measure aggregation, the aggregate stock diluted to 1 mg mL^‐1^ combined with the dye in a 96 well plate. The aggregate stock was spiked into unstressed IgG1 mAb A to generate different percentages of aggregates.

The four chosen dyes interact differently with protein aggregates (Fig. 1). They were also chosen as they solubilised in water, avoiding skewness in data due to the impact of buffers. To assess the potential of the four dyes for mAb aggregate detection in CHO cell cultures, first the dyes were verified in a purified system with thermally stressed mAb aggregates. The dyes were then applied to CHO cell culture supernatants of mAb producing and non‐mAb producing cell lines to see the implications of host cell protein on fluorescence.

In the literature, different conditions have been suggested to stress mAbs that can produce different amounts and types of aggregates. Kayser *et al*.^18^ stressed mAbs at 65 °C at 150 mg mL^‐1^ and He *et al*. used 50 °C for 8 h at 70 mg mL^‐1^ before SEC fractionation.^13^ Paul and Hesse described how heating at 65 °C formed larger aggregates greater than 1 µm.^17^ As the unfolding temperature for mAbs is above 65 °C^19^ it was decided to stress below this temperature to minimise fragmentation and formation of large aggregates. In addition, at higher concentrations >30 mg mL^‐1^, mAbs precipitated heavily when stressed. Therefore, for simplicity mAb A was stressed at the concentration achieved post‐purification.

### Detection of varying levels of purified mAb aggregates using dyes

**Figure 2:**
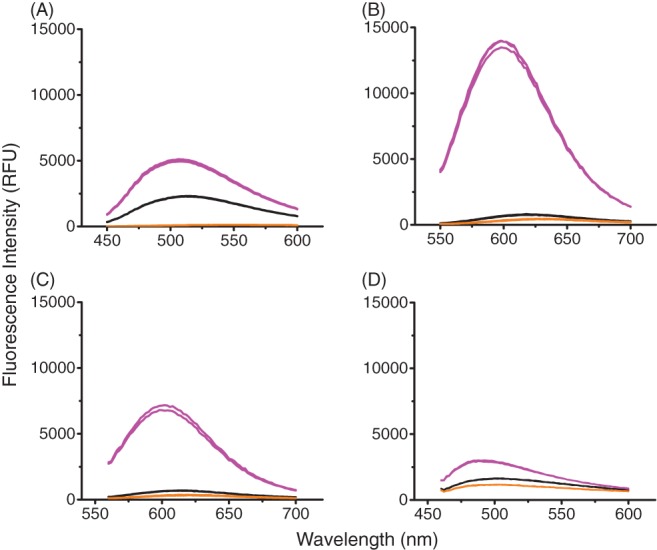
Fluorescence spectrum of dyes with mAb aggregates spiked into buffer. Thermally stressed IgG1 mAb A spiked into non‐aggregated mAb. Concentration of antibody in each well was 1 mg mL^‐1^. This was performed in triplicate. (A) 50 µmol L^‐1^ Bis‐ANS, ex/em‐ 390/450–600 nm, gain 70; (B) 5X SYPRO Orange, ex/em‐ 495/550–700 nm, gain 100; (C) 3 µM ProteoStat, ex/em‐ 530/560–700 nm; (D) 50 µmol L^‐1^ Thioflavin T, ex/em‐ 460–600 nm, gain 110. 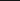 0% spiked aggregates 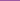 10 % spiked aggregates 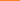 Buffer Blank**.**

**Figure 3:**
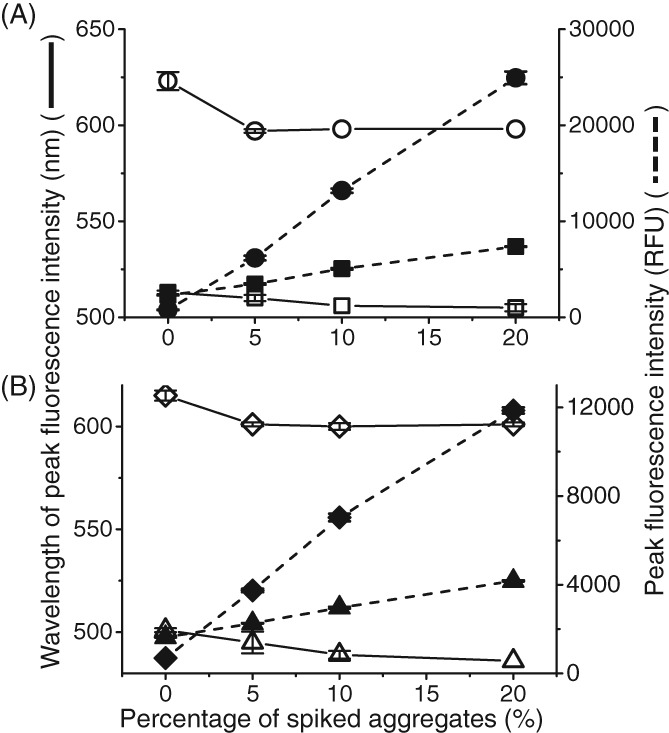
Peak height and peak wavelength against mAb A aggregates spiked into buffer. Thermally stressed IgG1 mAb A spiked into non‐aggregated mAb. Comparison of decrease in wavelength at which highest fluorescence occurs (degree of blue shift) against increasing fluorescence intensity with increasing amount aggregates. Concentration of antibody in each well was 1 mg mL^‐1^. This was performed in triplicate: (A) 50 µmol L^‐1^ Bis‐ANS, ex/em‐ 390/450–600 nm, gain 70 (wavelength SD = 1.63 nm, peak intensity SD = 78.4 RFU); 5X SYPRO Orange, ex/em‐ 495/550–700 nm, gain 100 (wavelength SD = 4.71 nm, peak intensity SD = 657 RFU); (B) 3 µmol L^‐1^ ProteoStat, ex/em‐ 530/560–700 nm (wavelength SD = 2.50 nm, peak intensity SD = 168 RFU); 50 µmol L^‐1^ Thioflavin T, ex/em‐ 460–600 nm, gain 110 (wavelength SD = 5.25 nm, peak intensity SD = 37.3 RFU). 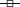 Bis‐ANS 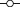 SYPRO Orange 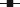 Bis‐ANS 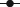 SYPRO Orange 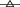 ProteoStat 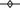 Thioflavin 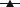 ProteoStat 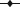 Thioflavin**.**

To first evaluate whether the dyes (Bis‐ANS, SYPRO Orange, ProteoStat and ThT) are capable of measuring aggregates in CHO cell culture supernatants, the dyes were first applied to a purified system. To measure the capability of the dye to distinguish different levels of aggregates, thermally stressed mAb A aggregates were spiked into a 96 well plate (Fig. 1). The stressed mAb A was measured by SEC to have 20% aggregates with a hydrodynamic radius of 22 nm. The aggregates were spiked into acetate buffer with unstressed monomeric mAb at varying amounts to provide samples with a 5–20% range of aggregation (Figs 2 and 3).

Figure 2 shows the spectral shape associated with each dye's emission with 10% mAb aggregates. As expected all four dyes had strong fluorescence intensities and clearly distinguished the different percentage of spiked aggregates, even as little as 5% aggregates (data not shown). There was little fluorescence in the buffer blanks (containing dye and buffer only) and good repeatability between replicates shape and peak fluorescence intensity.

Figure 3 shows the change in peak intensity wavelengths and the increase in fluorescence intensity with increasing amounts of aggregates. Notably all four dyes experienced a blue shift (a shift towards lower wavelengths) with increasing level of spiked aggregate. The highest spiked aggregate level (20%) had the greatest blue shift in all four dyes. A blue shift typically occurs due to an increase in the hydrophobicity around the dye.^20^ This can be a result of the increased exposure of hydrophobic residues to the solvent upon aggregation. Bis‐ANS and ProteoStat had similar degrees of blue shifts (6–8 nm) whereas SYPRO Orange and ThT had blue shifts greater than 10 nm. In these purified conditions, the fluorescence assay works well. To get a better understanding of how the assay would perform in a more complex environment, the dyes were next applied to the supernatants of mAb B and a null cell line.

### Application in CHO cell culture supernatants

When the fluorescent dyes were used in purified environments, they clearly distinguished different levels of aggregates with little fluorescence in the negative controls of buffer and unstressed mAb. However, there is the potential to have background fluorescence from the media when dealing with cell cultures. Paul *et al*. showed that the media has its own fluorescence after comparing media spiked with and without fluorescent dye (Bis‐ANS and ThT).^21^ They noticed similar fluorescence intensities in the media both with and without dyes. Cyclic and conjugated components present in the media can interfere with fluorescence measurements. This includes phenol red (a pH indicator and can cause significant quenching of fluorescence^22^) and riboflavin (excitation 450–490 nm and emission 500–650 nm) which overlaps with most of the dyes used in this study.^21^, ^22^, ^23^ In addition, some serum components, vitamins, amino acids groups and folic acid can be fluorescent.^24^, ^25^ However, in this study, phenol red, riboflavin and serum were not added into the media. Therefore, the fluorescence in the media was most likely caused by fluorescent amino acids and/or the dyes interacting with other protein, e.g. host cell proteins.

**Figure 4:**
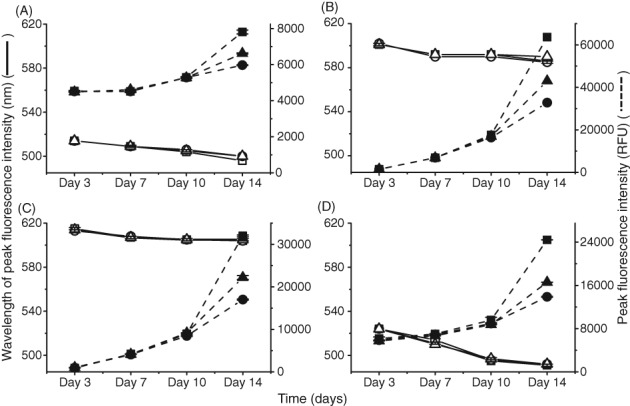
Comparison of change in peak fluorescence intensity and peak wavelength in three clarified null cell cultures shake flasks. Supernatants were separated by centrifugation and filtration before measuring adding dye and measuring fluorescence. Comparison of decrease in wavelength at which highest fluorescence occurs (degree of blue shift) against increasing fluorescence intensity with increasing amount aggregates. Concentration of antibody in each well was 1 mg mL^‐1^. This was performed in duplicate. (A) 50 µmol L^‐1^ Bis‐ANS, ex/em‐ 390/450–600 nm, gain 70 (wavelength SD = 1 nm, peak intensity SD = 108 RFU); (B) 5X SYPRO Orange, ex/em‐ 495/550–700 nm, gain 100 (wavelength SD = 1 nm, peak intensity SD = 603 RFU); (C) 3 µmol L^‐1^ ProteoStat, ex/em‐ 530/560–700 nm (wavelength SD = 1 nm, peak intensity SD = 449 RFU); (D) 50 µmol L^‐1^ Thioflavin T, ex/em‐ 430/460–600 nm, gain 110 (wavelength SD = 1 nm, peak intensity SD = 505 RFU). 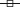 Shake Flask 1 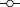 Shake Flask 2 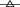 Shake Flask 3 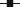 Shake Flask 1 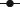 Shake Flask 2 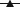 Shake Flask 3.

**Figure 5:**
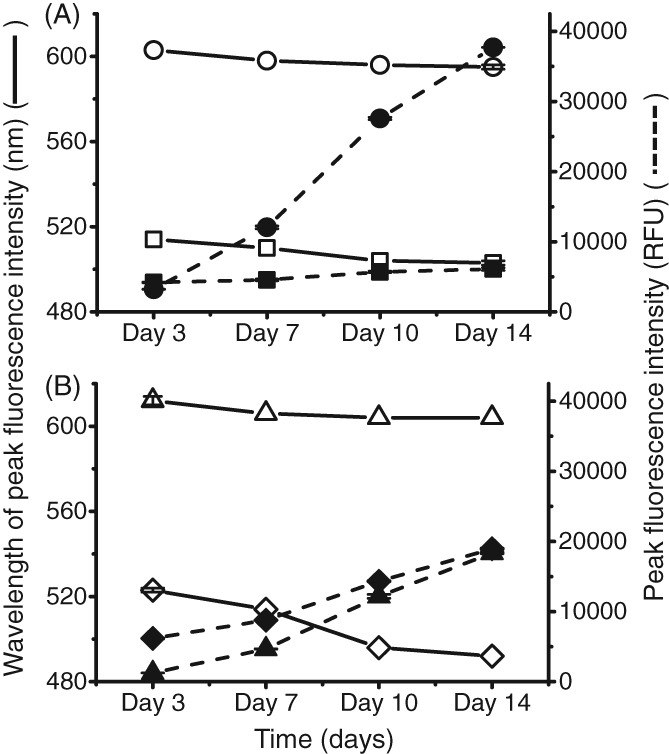
Peak height and peak wavelength against mAb clarified cell cultures. The dyes were spiked into wells containing clarified IgG1 mAb B from different time points. Supernatants were separated by centrifugation and filtration before measuring adding dye and measuring fluorescence. Comparison of decrease in wavelength at which highest fluorescence occurs (degree of blue shift) against increasing fluorescence intensity with increasing amount aggregates. Concentration of antibody in each well was 1 mg mL^‐1^. (A) 50 µmol L^‐1^ Bis‐ANS, ex/em‐ 390/450–600 nm, gain 70 (wavelength SD = 1 nm, peak intensity SD = 200 RFU); 5X SYPRO Orange, ex/em‐ 495/550–700 nm, gain 100 (wavelength SD = 1 nm, peak intensity SD = 207 RFU); (B) 3 µmol L^‐1^ ProteoStat, ex/em‐ 530/560–700 nm (wavelength SD = 2 nm, peak intensity SD = 278 RFU); 50 µmol L^‐1^ Thioflavin T, ex/em‐ 430/460–600 nm, gain 110 (wavelength SD = 1 nm, peak intensity SD = 122 RFU). 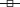 Bis‐ANS 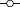 SYPRO Orange 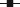 Bis‐ANS 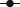 SYPRO Orange 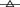 ProteoStat 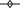 Thioflavin 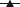 ProteStat 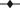 Thioflavin**.**

To better understand the contribution that HCPs may add to fluorescence, we investigated how the dyes interacted in a cell culture environment in the absence of the mAb (referred to as null cell culture). To do this, clarified null culture supernatant was used to evaluate the impact of HCP on fluorescence throughout the culture duration. The fluorescence of the dyes on different culture days of three null cell line shake flasks were compared with that of IgG mAb B (Figs 4 and 5). Each sample from the different days and shake flasks had the same concentration of dye added in each well.

#### *Null CHO culture*

**Figure 6:**
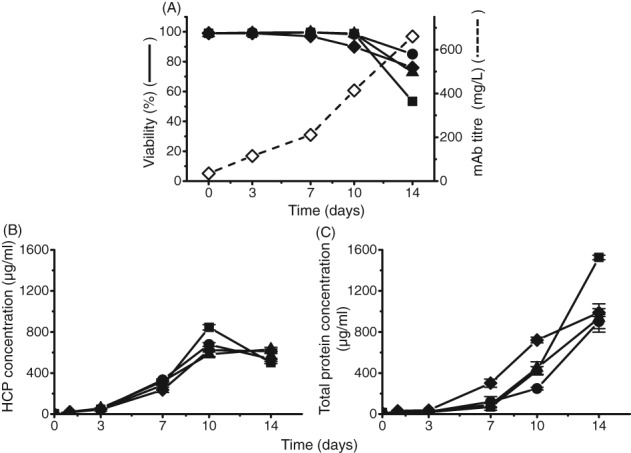
Protein concentration and viability measured in cell culture supernatants for null cell line and IgG mAb B. (A) Viability and mAb titre as measured by ViCell and Nephelometer respectively. (B) Host cell protein concentration over the 14‐day cell culture period as measured by in‐house ELISA (samples were serial diluted as necessary to fit into detection, SD = 26 µg mL^‐1^). (C) Total protein concentration as measured by Bradford assay (measured in triplicate. SD = 138 µg mL^‐1^). 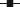 Null Shake Flask 1 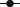 Null Shake Flask 2 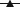 Null Shake Flask 3 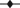 IgG mAb B 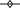 IgG mAb B titre.

In Fig. 4, all four dyes showed an increase in fluorescence intensity in the null culture supernatant samples over the duration of the cell culture. A trend seen among all four dyes was a steady and consistent rise in fluorescence intensity between day 3 and day 10. However, on day 14, to differing extents, a rapid increase in fluorescence intensity was observed. Bis‐ANS and ThT with the null culture supernatants showed an average increase in fluorescence intensity from day 10 to day 14 of 21% and 48%, respectively. ProteoStat and SYPRO Orange both showed an average increase in fluorescence intensity from day 10 to day 14 of 60%. The trend with fluorescence intensity among the three shake flasks on day 14 also correlated with loss of viability seen in Fig. 6(A). Shake flask 1 had the highest fluorescence intensity with the lowest viability out of the three shake flasks, whereas shake flask 2 had the lowest fluorescence intensity yet the highest viability on day 14. Among all four dyes, on average, fluorescence intensity of shake flask 1 sample was 22% higher than shake flask 2 on day 14.

It is well known that viability is a measurement of the number of cells that are alive (at the time of measurement). When cells die, the cell membrane becomes compromised, resulting in the exposure of intracellular components into the supernatant. As HCPs, can be hydrophobic, theoretically, HCPs can aggregate too with other HCPs, mAbs as well as other cellular components. This could possibly be being measured by the dye assay. Looking at the HCP concentration over the 14‐day culture (Fig. 6(B)), there was a decrease from day 10 to day 14 in HCP concentration for shake flask 1 and 3. For shake flask 2 there was more of a plateau. However, looking at the total protein concentration (Fig. 6(C)), we clearly saw that null shake flask 1 has 40% higher total protein concentration than that of shake flask 2 and 3. There was also a blue shift in lambda max seen from all four dyes which refers to the dye being in a more hydrophobic environment, similarly seen in Fig. 3. Between day 3 and day 14 of the null culture samples, for Bis‐ANS, SYRPO Orange and ThT there was a 2–3% decrease in peak wavelength. For ThT there was a greater decrease in peak wavelength of the null culture samples between day 3 and day 14 of 7%.

#### *mAb B CHO culture*

In Fig. 5, all four dyes also showed a linear increase in fluorescence intensity in the IgG mAb B culture supernatant samples over the duration of the cell culture. Comparing the fluorescence intensities between the mAb and null culture supernatants, the mAb had similar intensities to null shake flask 3 with all four dyes. This also correlated with viability as both null shake flask 3 and the mAb culture supernatant on day 14 shared similar viabilities. HCP concentration (Fig. 6(B)) and total protein concentration (Fig. 6(C)) of null culture shake flask 3 was also similar to IgG mAb B. This could indicate that both samples had similar levels of aggregation. Based on this trend with fluorescence intensity, viability and total protein, it explains the difference between null culture shake flask 1 (Fig. 4) and IgG mAb B (Fig. 5) on day 14. Null culture shake flask 1 had a lower viability (Fig. 6(A)) and higher total protein concentration (Fig. 6(C)) than the IgG mAb B on day 14. Hence, null culture shake flask 1 had a higher fluorescence intensity which may suggest a higher presence of aggregates.

With all four dyes, a blue shift with the IgG mAb B culture supernatants was also seen. Between day 3 and day 14 of the null culture samples, Bis‐ANS, SYRPO Orange and ThT had a 1–2% decrease in peak wavelength. For ThT, there was a greater decrease in peak wavelength of IgG mAb B culture samples between day 3 and day 14 of 8%. However, the strong fluorescence intensities seen in the null cultures supernatants in the presence of dyes and the absence of mAb, shows that HCP must strongly influence fluorescence of these extrinsic dyes. One possible reason could be that the dyes are interacting with secreted endogenous produced proteins as well as intracellular proteins exposed to the supernatant following apoptosis at low viabilities.

Overall, this emphasised that even in the absence of mAb and irrespective of culture age that extrinsic dyes are not a specific indicator of mAb aggregation. However, they may rather be an indicator of overall protein aggregation or high molecular weight species.

### Characterisation of the size of aggregates the dyes are interacting with

Aggregates differ by size and shape depending on the mechanism of aggregation. As we showed that the extrinsic dyes were not a specific indicator of mAb aggregation, we wanted to gain insight into what size and type of proteins the dyes may be interacting with. Focusing on SYPRO Orange and ProteoStat, we investigated null cell culture and IgG mAb B supernatants to see whether the dyes interact more with high molecular weight (HMW) or low molecular weight (LMW) or both. We focused on SYPRO Orange and ProteoStat as they were less protein concentration dependent and had different mechanisms of interacting with the aggregates (hydrophobicity vs molecular rotor).

**Figure 7:**
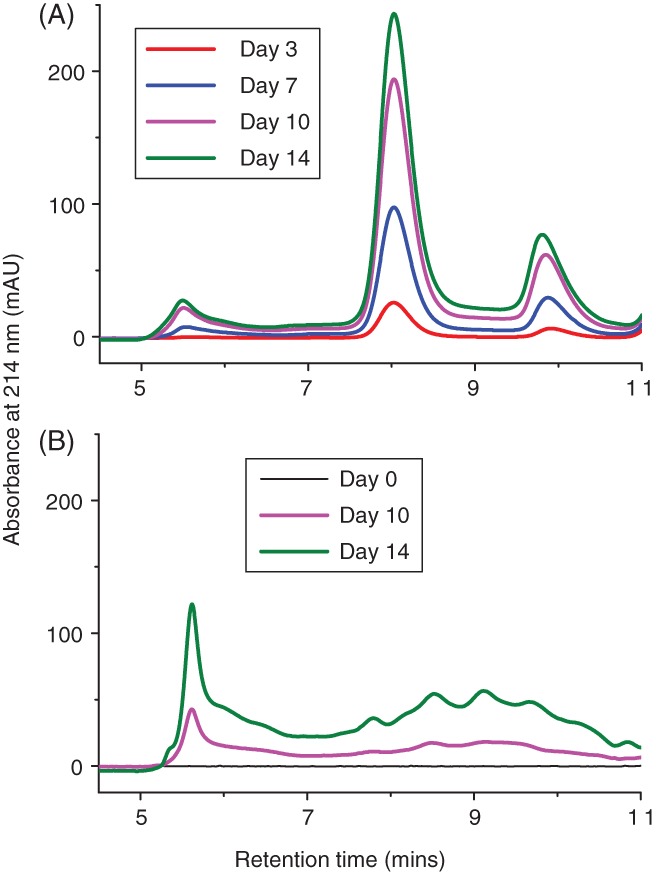
SEC of IgG1 mAb B and null cell line culture supernatants. (A) IgG mAb B and (B) null cell line shake flask 1were analysed using size exclusion chromatography to understand the aggregation profile directly in culture. The samples 0.22 µm filtered before running on TSKgel2000SWI column (7.8×300mm) with a flow rate of 0.5 mL min^‐1^. The detector measured absorbance at 214 nm. Running buffer composed of 100 mmol L^‐1^ sodium phosphate (monobasic), 400 mmol L^‐1^ sodium chloride, pH 6.8.

Clarified supernatants of the null culture (shake flask 1) and IgG mAb B were applied on to SEC (Fig. 7) to assess the profile of LMW to HMW species at 214 nm. Based on the column specifications, species eluting before 5 min were greater than 500 kDa and after 11 min were smaller than 10 kDa. Figure 7 shows an increase in peak area from day 0 to day 14 for both the null and mAb culture supernatants, indicating an increase in the concentration of HMW and LMW species over time. For the null culture, there was a sharp peak after the void volume showing a high amount of HMW. This peak is also seen in the mAb B supernatants, but is not as intense.

**Figure 8:**
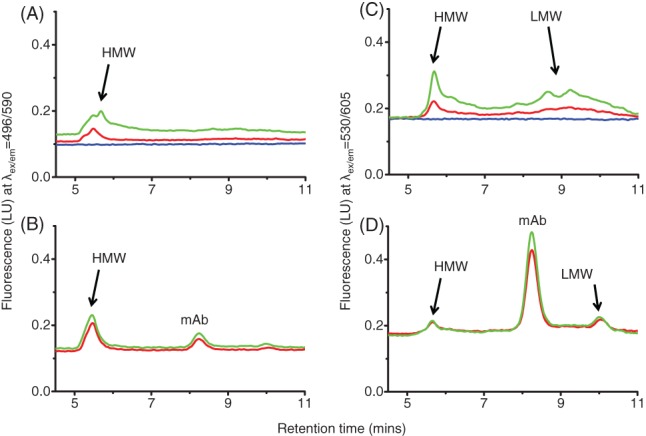
SEC of mAb and null clarified cell cultures with SYPRO Orange and ProteoStat. (A) Null cell line shake flask 1 with 5× SYPRO Orange; (B) IgG mAb B with 5× SYPRO Orange; (C) null cell line shake flask 1 with 3 µmol L^‐1^ ProteoStat; (D) IgG mAb B with 3 µmol L^‐1^ ProteoStat. Clarified cell culture samples were combined with dye before loading onto the column. A TSKgel2000SWI column (7.8×300 mm) with a flow rate of 0.5 mL min^‐1^ with a fluorescence detector was used. SYPRO Orange samples ex/em 495/590 nm, ProteoStat samples ex/em 530/605 nm. Running buffer composed of 100 mmol L^‐1^ sodium phosphate (monobasic), 400 mmol L^‐1^ sodium chloride, pH 6.8. Injection volume of 50 µL. 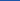 Media 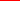 Day 10 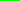 Day 14**.**

To get an indication of the size of protein species that the dyes were binding to, null culture shake flask 1 and IgG mAb B were mixed with SYPRO Orange or ProteoStat prior to injecting onto the SEC column with fluorescence detectors. The IgG mAb B and null culture with SYPRO Orange in Fig. 8(A) and (B) had strong HMW peaks at around 5.5 min. This indicated the presence of aggregates which we already know are present from the UV traces in Fig. 7(A) and (B) at 5 min. The IgG mAb B culture with SYPRO Orange (Fig. 8(B)) also notably detected the monomer mAb species at around 8 min.

For the null culture with ProteoStat in Fig. 8(C), there was a strong HMW peak at around 5.5 min. We also saw that null culture with ProteoStat (Fig. 8(C)), there was a stronger increase in fluorescence signal of LMW species at 9 min compared with the null culture with SYRPO Orange (Fig. 8(A)). There was also a more defined LMW species peak seen with IgG mAb B (Fig. 8(D)) with ProteoStat at 10 min than with SYPRO Orange (Fig. 8(B)). The IgG mAb B culture with ProteoStat (Fig. 8(D)) had a surprisingly strong monomeric mAb signal at around 8 min. This signal was stronger than both the HMW and LMW. ProteoStat also detected the HMW in the null culture on day 14 (Fig. 8(C)) with higher intensities than the IgG mAb B on day 14 (Fig. 8(D)). This correlated with the plate dye assay whereby we saw that the null culture shake flask 1 had higher fluorescence on day 14 than the IgG mAb B.

This led to the conclusion that SYPRO Orange was more sensitive at detecting very large molecular weight species. ProteoStat on the other hand, was not as effective as SYPRO Orange at measuring very large aggregates and is perhaps better suited to smaller aggregates. In addition, ProteoStat binds to monomeric mAb which may skew fluorescence results compared with SYPRO Orange.

## CONCLUSION

A current challenge in bioprocessing is the ability to measure critical quality attributes without prior purification. Fluorescent dyes have been known for their sensitivity and ability to measure aggregates based on different types of interactions. With the dyes used in this study, the main interactions with aggregates were due to interacting with exposed hydrophobic regions on proteins (Bis‐ANS and SYPRO Orange), and constrained dye rotation resulting in fluorescence (ThT and ProteoStat). All four dyes used in this study worked well in purified conditions, however, there has been limited application of fluorescent dyes in cell culture. The only previous study showed Bis‐ANS to be suitable to analyse mAb aggregate from cell cultures. However, from this study, we showed an increase in fluorescence in both null and mAb clarified cultures in the presence of dyes from inoculation up to day 14. The fluorescence in the null clarified supernatant had similar (and in one case higher) fluorescence intensities to/than the mAb clarified supernatant. This showed that fluorescent dyes solely are not a specific indicator of mAb aggregation. However, the fluorescent dyes used in this study revealed the potential to use as not only an indicator of protein aggregation but also screening for viability. This study also provided interesting insight into the type of aggregates the dyes are interacting with. It seemed that SYPRO Orange is more sensitive for measuring large aggregates whereas ProteoStat is able to detect aggregates, monomeric mAb and fragments to similar extents. One potential application of SYPRO Orange may be to highlight cell lines which may produce large aggregates during the cell culture which may cause issues during harvest. It could be used to aid cell line selection in maximising viabilities and minimising the amount of aggregates.
